# A Case in Which 16 Sewing-Needles and 4 Pins Were Extracted from the Wrist

**Published:** 1845-04

**Authors:** 


*A case in which sixteen Sewing-Needles and four Pins were extracted from the Wrist.*—About the 21st or 22d of September last, I was called to see a young lady, Miss P———, with her hand and wrist much swollen and inflamed, and very painful and stiff, in consequence, as she supposed, of a sprain. The pain was of the acute lancinating kind, and was much increased by the least attempt to move or bend the wrist. She was ordered to keep it perfectly at rest and apply evaporating lotions.

Sunday, September 29th, called again, and found the hand and wrist of nearly normal appearanee, but she was not able to bend the joint without violent pain, extending up the whole length of the arm. On examination, I discovered on the back of the wrist, a short distance below the extremities of the radius and ulna, and near the middle of the wrist, just under the skin, some foreign body, apparently confined between the bones. Supposing from her account that it must be a small spiculum of bone, I made an incision directly over the body, and having by a probe ascertained its exact position, I introduced a small pair of forceps, and, to my great surprise, extracted three pieces of needles. During the ensuing week, at different times, I extracted other pieces, in all eleven whole needles and four pins without heads. Since that time, my father, Dr. Gordon P. Spencer, has extracted and sent to me two others. In all sixteen needles and four pins have been extracted—three of the needles were broken into numerous pieces, all were much corroded, and most of them considerably bent. How these bodies came there, it is impossible to say, but from the manner in which they were confined, the depth from which they were taken, and the whole appearance of the parts as well as of the needles, it seems impossible that she could have voluntarily introduced any one that was extracted. Some were found as much as two inches above the incision, laying directly between the bones.

*Medical Examiner.*

The editor decides upon this case, no doubt correctly, that the young lady herself inserted these instruments—probably during a temporary aberration of mind, known not to be a rare occurrence in hysterical females. How else could the pins and needles have found their way into such a place?

